# A study of Samoan, Tongan, Cook Island Māori, and Niuean infant care practices in the Growing Up in New Zealand study

**DOI:** 10.1186/s12889-024-17680-1

**Published:** 2024-01-12

**Authors:** Fiona C. Langridge, Janine Paynter, Luam Ghebreab, Maryann Heather, Amio Matenga-Ikihele, Teuila Percival, Vili Nosa

**Affiliations:** 1 Department of Paediatrics: Child and Youth Health, School of Medicine, Faculty of Medical and Health Sciences, The University of Auckland, Auckland, New Zealand; 2 Department of General Practice and Primary Healthcare, School of Population Health, Faculty of Medical and Health Sciences, The University of Auckland, Auckland, New Zealand; 3 Pacific Health Section, School of Population Health, Faculty of Medical and Health Sciences, The University of Auckland, Auckland, New Zealand; 4 Moana Connect, Māngere, Auckland, New Zealand

**Keywords:** Sudden infant death, Pacific, Risk factors

## Abstract

**Background:**

Despite a low rate of infant mortality, Aotearoa New Zealand has a high rate of Sudden Unexpected Death in Infants (SUDI), with disproportionate impact for Pacific infants. This study explored the infant care practices, factors and relationships associated with increased risk of SUDI amongst Tongan, Samoan, Cook Islands Māori, and Niuean mothers in New Zealand, to inform evidence-based interventions for reducing the incidence of SUDI for Pacific families and their children.

**Methods:**

Analysis comprised of data collected in 2009–2010 from 1089 Samoan, Tongan, Cook Islands Māori and Niuean mothers enrolled in the Growing Up in New Zealand longitudinal cohort study. The sleeping environment (bed-sharing and sleep position) of the infants was assessed at 6 weeks. Multivariable logistic regression analysis were conducted, controlling for sociodemographic factors to explore the association between selected maternal and pregnancy support and environment factors and the sleeping environment for infants.

**Results:**

Mothers who converse in languages other than English at home, and mothers who consulted alternative practitioners were less likely to follow guidelines for infant sleeping position. Similarly language, smoking, alcohol, household dwelling, crowding and access to a family doctor or GP were associated with mothers following guidelines for bed-sharing.

**Conclusion:**

The impact of SUDI on Pacific infants may be lessened or prevented if communication about risk factors is more inclusive of diverse ethnic, cultural worldviews, and languages. Societal structural issues such as access to affordable housing is also important. This research suggests a need for more targeted or tailored interventions which promote safe sleeping and reduce rates of SUDI in a culturally respectful and meaningful way for Pasifika communities in Aotearoa, New Zealand.

## Background

Sudden unexpected death in infancy (SUDI) is a broad term that describes the initially unexplained death of an infant under the age of one [1–4]. It includes deaths in circumstances of high risk, such as when an infant dies due to suffocation or asphyxiation due to bed sharing or soft bedding, or an infection, or previously unrecognised genetic, cardiac or metabolic anomaly (‘explained’ SUDI) as well as sudden infant death syndrome (SIDS) [5].

Globally SUDI remains a leading cause of infant death in high-income countries, even after dramatic decreases during the 1990s after the instigation of public health campaigns related to sleeping infants on their backs and avoiding co-sleeping [6–8].

The New Zealand Cot Death Study in the 1990s identified key risk factors including a baby sleeping on their front, maternal smoking, not being breastfed and bed sharing [5, 9–12]. Further investigation has shown a much higher risk of death with the combination of maternal smoking in pregnancy and bed sharing after birth [13]. Alcohol is a risk and that risk increases when the mother consumes alcohol and bed shares [13–15]. Infants who share their sleeping room at night with one or more adult have a lower risk of SUDI than those that do not share [9]. Immunisations have been associated with halving the risk of SIDS suggesting vaccine preventable disease (VPD) increases SUDI risk or both SUDI and VPD co-occur frequently due to deprivation [16].

A thematic analysis of SUDI liaison reports in Aotearoa New Zealand from 2018 to 2020 found the following factors were raised in the reports: Extreme maternal and paternal tiredness leading to co-sleeping, caring for an unwell infant, non-parental caregivers, overcrowding, maternal mental wellbeing and housing availability and affordability [5].

Despite a relatively low rate of infant mortality, Aotearoa New Zealand carries one of the highest rates of SUDI, with disproportionate impact for Māori and Pacific infants [4, 17, 18]. After risks for SUDI were identified in the 1990’s, public health campaigns reduced the SUDI rate in Aotearoa New Zealand from 4.45 per 1000 live births in the late 1980’s to 0.9 per 1000 live births in 2015 [18]. Aotearoa New Zealand has not achieved the national goal of 0.1 deaths per 1000 reached by other Organisation for Economic Co-operation and Development (OECD) countries [5].

The Ministry of Health in Aotearoa New Zealand have funded a national programme for delivery of safe sleep messages, and provision of safer sleep spaces since 2017. Safe sleep messaging includes: ensuring the baby is in their own bed for every sleep (and in the same room as the adult looking after them for the night), and making sure the baby is on their back for every sleep [19]. Provision of safe sleeping spaces have included Wahakura (woven flax basket), a traditional Māori bed for babies, and the pēpi-pod (a small cot made with clear plastic that can contain a sleeping baby on a bed next to parents), for reducing risk associated with bed-sharing while maintaining physical closeness of baby and caregiver [20]. This resulted in improvements in post-perinatal deaths between 2010 and 2015, however these improvements have now plateaued [17]. Rather there are some statistics suggesting a significant increase in rates, especially for Māori. The death rate for Pacific infants remains higher than those for non-Māori and Other ethnicities since 2002, but lower than for Māori [5]. In 2022 it was reported Pacific infants are six times more likely (RR 5.85) to experience sudden infant death than non-Māori, non-Pacific infants [17]. In 2000, Pacific led research investigated SUDI deaths amongst Pacific peoples and found some Pacific SUDI deaths had not been attributed correctly as Pacific SUDI deaths, highlighting inaccuracies in collection and reporting of ethnicity data [21].

It is thought the impact on Pacific infants may have been lessened or prevented if communication about risk factors had been more inclusive and thoughtful about diverse ethnic and cultural worldviews, and if there was no inequity in smoking prevalence. The need for culturally appropriate interventions and Pasifika workforce for Pacific communities is still pressing [17, 22, 23]. Risk factors such as cigarette smoking are widely recognised as a marker of deprivation and are therefore difficult to change without concerted focus on the social determinants of health which also disproportionately impact Pasifika communities [17]. Despite the need for a more targeted response there has been little research to provide evidence for effective interventions to improve rates of SUDI for our Pasifika communities in Aotearoa, New Zealand.

In response this study aims:

- To describe the infant care practices relating to risk of SUDI amongst Tongan, Samoan, Cook Islands Māori, and Niuean mothers in Aotearoa New Zealand.
- To describe health care service components and socio-cultural and demographic factors associated with safe sleeping arrangements for infants.

## Methods

### Study participants and data source

This study used data of participants enrolled in the longitudinal birth cohort Growing up in New Zealand (GUiNZ). The details of the GUiNZ study design, recruitment and the main characteristics of the cohort have been discussed previously [24, 25]. 6822 pregnant mothers were recruited before birth with the expected delivery dates from April 2009 to March 2010 from three defined geographical regions broadly generalisable to the current population of New Zealand births. The children of these mothers form the GUiNZ cohort. Care was taken in selecting the study region to ensure adequate enrolment of Māori and Pacific children [26]. A total of 6846 live births made up the participant cohort for GUiNZ [25]. Multiple age-appropriate and child development domain-specific data collection waves (DCWs) were conducted to date, using computer-assisted personal face-to-face interviews, telephone interviews and data linkage.

**Fig. 1:**
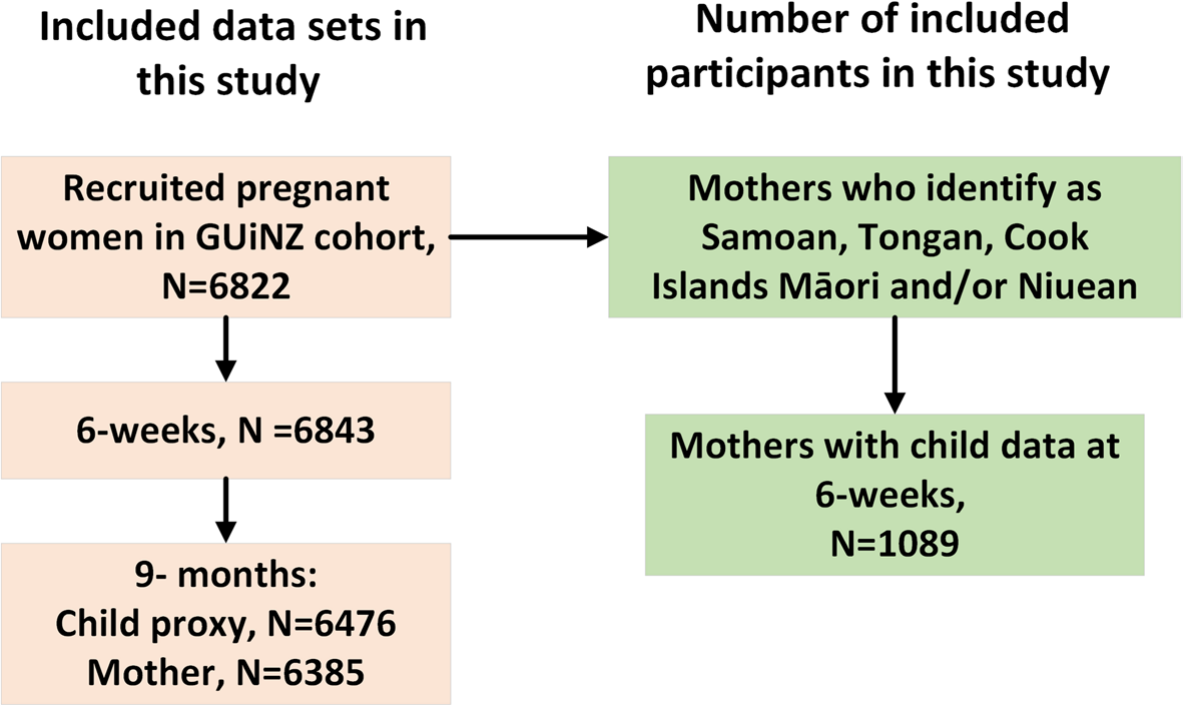
Flowchart of the data sets included, and the final Pacific mothers included in this study

The current study included children of mothers who identify with Pacific ethnicities during the antenatal DCW. GUiNZ study gathered ethnicity-related data using multiple levels of Statistics New Zealand categories for ethnicity [27]. A subset of the longitudinal cohort for the Pacific was created based on the Statistics New Zealand classification of Ethnicity Level 4, which is more detailed and provides disaggregated Pacific peoples ethnicity - Samoan, Tongan, Cook Islands Māori, and Niuean. These ethnicities were selected as this quantitative study was intentionally designed to inform a qualitative study focussing the four largest Pacific groups in New Zealand. Figure 1 shows a flowchart describing the datasets included, and the final Pacific mothers included in the study. Pacific peoples making up > 98% of the total Pacific population living in New Zealand [28].

### Measures

The measures used in this study are derived from data gathered at three-time points -antenatal maternal data, perinatal interview data and (6- weeks postnatal) -antenatal interview, and 9-months maternal and child data. During the antenatal interview, *N* = 1108 mothers identified as Samoan, Tongan, Cook Islands Māori and Niuean Pacific or combinations of these ethnicities.

#### Outcome of interest

The infant care practices were assessed when children were 6 weeks old during DCW 1. The sleeping environment, such as sleeping in a separate room to an adult, and sharing beds with another person or co-sleeping, was assessed using multiple choice and including an ‘other’ option for an open response. The responses were dichotomised as ‘Not meeting sleep arrangement guidelines’ (if a child sleeps in parents’ bed in a protected space, in parent’s bed not in a protected space, in an infant cot in a room alone, or in a separate room with siblings) vs “Meeting sleep arrangement guidelines” (infant cot in parents room) [29]. Sleeping position is another important infant care practice assessed as an outcome variable related to increased risk of SUID. The non-supine or prone sleeping positions are associated with a higher risk of SUDI [30] thus categorised as ‘not safe’ sleep positions, and the supine infant sleep position as ‘safe’.

#### Demographic

Demographic and maternal factors that are potentially associated with the infant care practices such as maternal age, highest educational attainment, and parity were included in the analysis of this study. Maternal age distribution was assessed using five-year categorisation; it was then dichotomised (< 30 years vs. ≥30 years) when included in the regression analysis. The maternal educational qualifications were initially classified according to measures from the Statistics New Zealand’s national census [31]. In this current study, the maternal education had three categories (No secondary school qualification/Secondary School, Diploma/Trade Cert (NCEA 5–6) and Bachelor’s or Higher degree) while parity was assessed using discrete numbers.

#### Exposures

##### Maternal mental health and lifestyle

During the antenatal data collection wave, maternal depression was assessed using the Edinburgh Postnatal Depression Scale (EPDS) [32]. The total score out of 10-item questions (max total score = 30, with each item score 0–3) was calculated and dichotomised into maternal depression versus no depression based on the cut-off point 13, which is less sensitive but more specific [33]. Individuals with a score of 13 or greater are considered to have significant antenatal depressive symptoms. At this cut-off, the EPDS has reported sensitivity and specificity for major depression in pregnancy of 0.83 and 0.90; respectively. Mothers were also asked to provide information about smoking and alcohol consumption status both during the antenatal DCW and at 9-months DCW. The categorisations were based on the time of consumption, amount, and maintaining a reasonable number of counts within each subgroup.

##### Family and home context

Area-based socioeconomic deprivation was measured within the GUiNZ longtidinal study using nine variables to derive the New Zealand Index of Deprivation (NZDep) [34]. The NZDep index is usually displayed in deciles from 1 as the least derived score to 10 most deprived score. GUiNZ use the deprivation scores to derive a variable grouped into three categories: low deprivation (deciles 1–3), which were the least deprived; medium deprivation (deciles 4–7), and high deprivation (deciles 8–10), which were the most deprived. Other aspects of household information such as housing ownership (owners vs. public/privately rented) and crowding index [35] categorised as low; < 2 people per bedroom, medium; between two and three people per bedroom and high; > 3 people per bedroom were determined.

##### Community and cultural context

An index was created for cultural connectedness using five 5-pt likert scale variables including cultural knowledge (1. Very knowledgeable, 2. Fairly knowledgeable, 3. Somewhat knowledgeable, 4. Not very knowledgeable and 5. Not at all knowledgeable), involvement in traditional cultural activities (1. Very involved, 2. Fairly involved, 3. Somewhat involved, 4. Not involved much, 5. Not involved at all), feelings toward their culture (1. Very positive, 2. Fairly positive, 3. Neither positive nor negative, 4. Slightly negative and 5. Very negative), frequency of association with others in the ethnic group (1. Most of the time, 2. Often, 3. Sometimes, 4. Not Often, 5. Almost never) and importance of maintaining cultural practices (1. Very important 2. Fairly important, 3. Somewhat important, 4. Not very important, 5. Not important at all). Each variable was dichotomised by combining the 1 and 2, and then 3–5. The five variables were summed with a score of 4–5 = “Very connected”, 2–3 = “Moderately connected, 0-1 = “Poorly connected”.

A second index was created for amount of family support using five 6-pt likert scale variables, including support from partners, parents, in-laws,extended families and partners extended family (1. Not available, 2. Not at all helpful, 3. Sometimes helpful, 4. Generally helpful, 5. Very helpful, 6. Extremely helpful). Each variable was dichotomised by combining 1–2 and 4–6.The total score was a sum of those variables, Very supported (3–5) and Poorly supported (0–2). GUiNZ cohort has diverse families; one-third of the involved parents had at least one parent overseas [25]. Thus, a wide range of languages was being spoken by parents at home, and this study dichotomised their language spoken at home as English or not English.

##### Healthcare access and preparation

With respect to healthcare access, information regarding regular primary healthcare access during their pregnancy including type of Lead Maternity Carer (LMC), the duration to find access and if their newborn will have the same general practitioner (GP) as the mothers were sought and included in this analysis. Further information was analysed on the mother’s intention to immunise and childbirth preparation course and if they have plans to be seen by a complementary or alternative practitioner.

##### Other SUDI protective/risk factors

All mothers in an antenatal stage in the GUiNZ study were asked whether they intended to breastfeed their babies after being born with 6-week phone call interviews to follow up on whether these intentions for feeding were attained. This study included the self-reported feeding responses at 6- weeks which were five answer options of: Breast milk only, Mainly breast milk, but has also received some water based drinks, Formula only, Formula and breast milk or Other, Please specify. Gestational age at delivery is an important predictor of immediate perinatal health, including SUDI; as such, prematurity (Full term/premature) was assessed in this study.

### Statistical analysis

Prior to the commencement of data analysis, data were explored for duplicates and the extent of missed data in each variable included in this study using SAS and R programming software. Initially, descriptive analysis of the infant care practices (sleeping arrangements), demographic distribution, health access, maternal health and lifestyle, family, community, and other social factors were conducted for each disaggregated Pacific ethnicity included in this study. The analysis was presented as proportions and frequencies for categorical variables and means and standard deviation or median and standard deviations when the variable of interest is continuous depending on the normality of distribution. This was followed by the preliminary univariate analysis using the binary logistic regression to determine the factors associated with infant care outcomes by comparing the Pacific mothers who practised the recommended guidelines of sleeping arrangements and positions versus those who did not. The multicollinearity of significant explanatory variables was further carried out using generalised variance inflation factors; the rule of thumb for removing one of the redundant variables with multicollinearity concern is usually greater than four [36].

The multivariable logistic regression models were fitted to estimate the association of significant exposures related to infant care practices. In the multivariable model, all the variables were controlled for maternal education, deprivation and maternal age to identify the factors affecting the recommended infant sleeping position and safe sleeping arrangements as per the guidelines.

## Results

Of the total 6822 interviewed mothers during the antenatal DCW, while pregnant, 1108 were selected as study participants in this study (Samoan (47%, *n* = 516), Cook Island Māori (21%, *n* = 233), Tongan (27%, *n* = 317) and Niuean (9%, *n* = 108)) (Table 1). The total percentage adds up to more than 100% because there were mothers who identified with more than one of these four ethnicities included.

**Table 1:** Demographics of the Pacific mothers and their infants (*N* = 1108)

GENERAL DEMOGRAPHICS	Total		Samoan		Cook Island Māori		Tongan		Niuean	
	N	(%)	N	(%)	N	(%)	N	(%)	N	(%)
Total	1108		516	47.0	233	21.0	317	29	108	10
Age of parent - antenatal										
Missing	0	0								
< 20	106	10	46	9	29	12.0	24.0	8	20	19
20–24	279	25	124	24	83	36.0	61	19	29	27
25–29	291	26	126	24	56	24.0	92	29	31	29
30–34	232	21	115	22	35	15.0	79	25	18	17
35–39	159	14	78	15	26	11.0	50	16	10	9
> =40	41	4	27	5	< 10	2.0	11	3	0	0
Highest completed qualification										
Missing	< 10		< 10	0			< 10	0	< 10	1
No secondary school education	154	14	48	9	54	24	42	13	24	22
Secondary school NCEA levels 1–4	449	41	214	41	79	34	143	45	37	34
Diploma trade/NCEA levels 5–6	392	35	197	38	85	36	93	30	35	32
Bachelor’s degree	79	7	38	7	< 10	4	29	9	< 10	7
Higher Degree	30	3	17	3	< 10	2	< 10	3	< 10	3
Number of children										
Missing	129	12	56	11	35	15	32	10	10	9
0	306	28	148	29	56	24	79	25	48	44
1	225	20	113	22	48	21	64	20	17	16
2	169	15	78	15	40	17	50	16	11	10
3	115	10	57	11	27	12	27	9	< 10	6
4 or more	164	15	64	12	27	12	65	21	15	14
Prematurity										
Missing	19	2	< 10	2	< 10	1	< 10	2	< 10	4
Full Term	1030	93	485	94	219	94	287	91	100	92
Premature	59	5	22	4	12	5	23	7	< 10	4
Deprivation										
Missing	139	13	65	12	29	12	45	14	12	11
Low	40	4	15	4	11	5	11	3	< 10	4
Medium	163	15	84	13	26	11	49	15	16	15
High	766	69	352	70	167	72	212	67	76	70

**Fig. 2:**
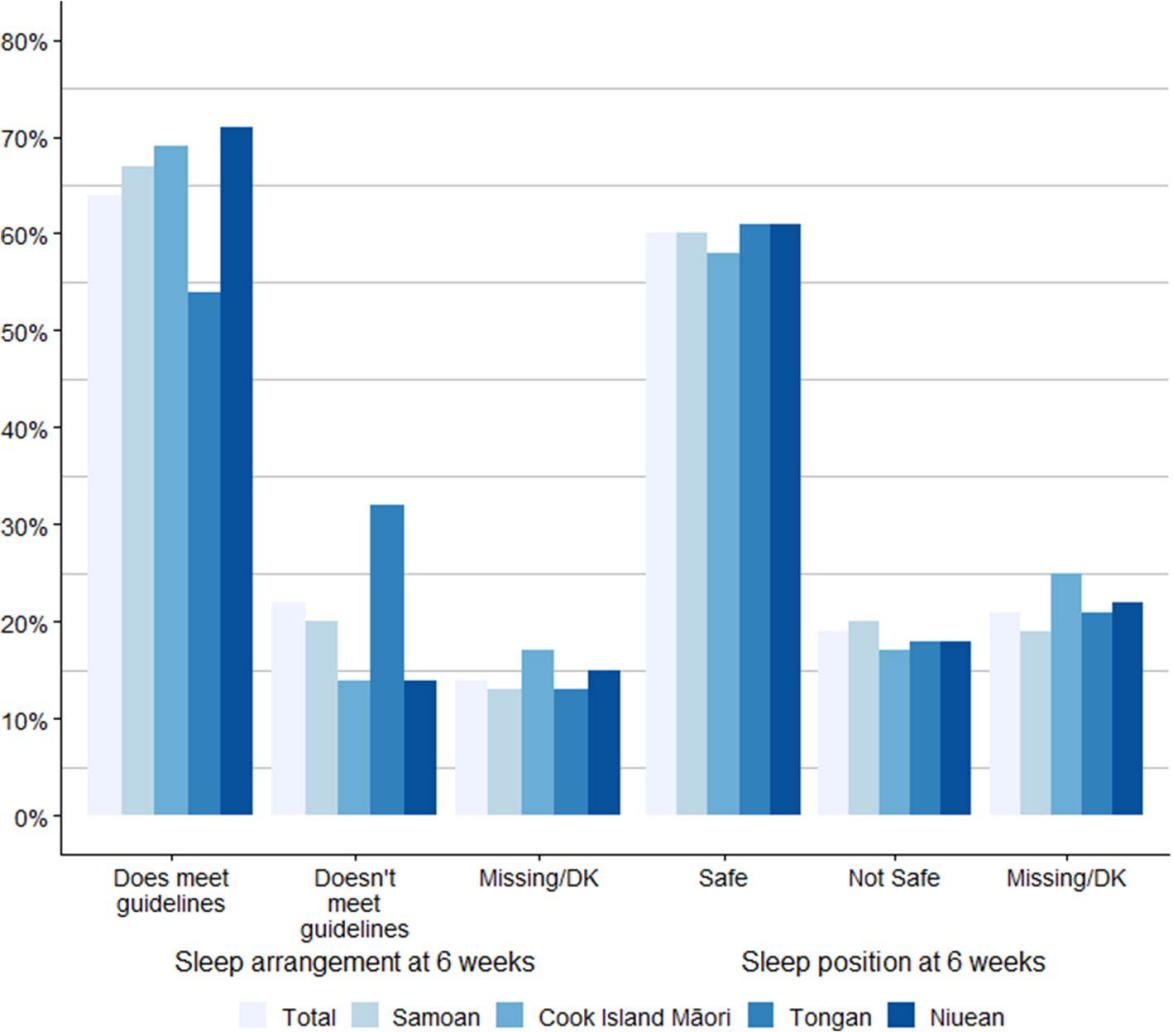
Infant care practices among Pacific families

At approximately 6 weeks, 64% (*n* = 705/1108) of mothers reported their babies were sleeping in a cot or bassinet in their parents’ room, meeting the infant sleep arrangements guidelines, whereas 22% (*n* = 245/1108) were sleeping in an infant cot/bassinet but in different rooms and sleeping in their parent’s bed with or without defined space (Fig. 2). Mothers also reported their infant’s sleep position in their first few weeks of life, 60% (*n* = 668/1108) were sleeping on their backs which the Ministry of Health recommends; 19% (*n* = 207/1108) were sleeping either on their stomach or back and the remaining 21% information was missing or “do not know” response. The infant care practices were similar among all the pacific ethnicities included, but fewer Tongan mothers (54%) met the sleep arrangement guidelines compared to others, in this cohort, ranging from 67 to 71%.

**Fig. 3:**
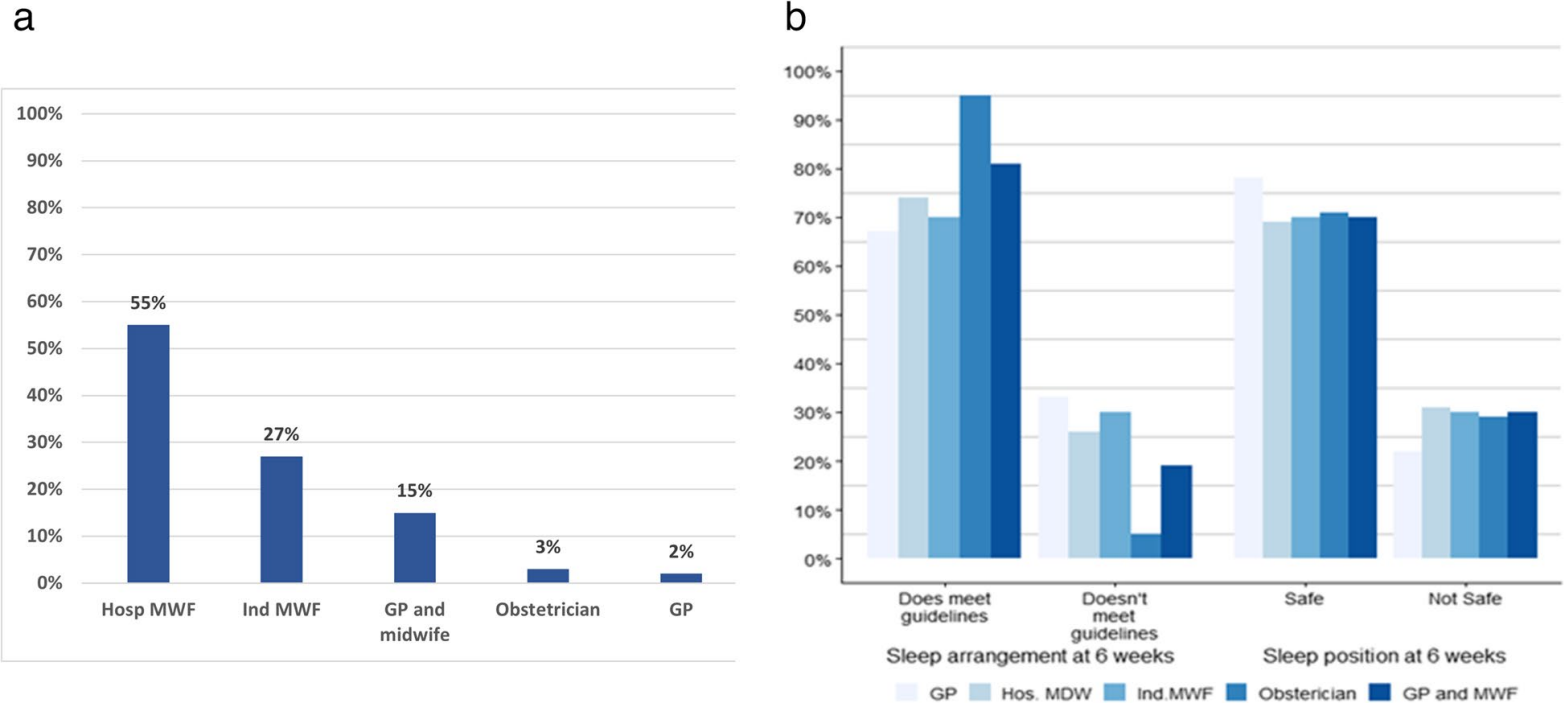
**a** and **b**. Type of lead maternity caregiver LMC, Hosp MWF is hospital midwife, Ind MWF is independent midwife, GP is general practitioner, MWF is midwife

The type of LMC that the participant accessed is summarised in Fig. 3a. More than half (55%) of all mothers were cared for by a hospital-based midwife (Hosp MWF), followed by independent midwives (Ind MWF) (27%) and combinations of both general practitioner and midwives (15%). The least used type of carer in pregnancy were obstetricians (3%) and general practitioners (2%). Figure 3b summarises the proportion of types of LMC accessed by infant care practice. The only notable difference in sleep arrangement guidelines was the high proportion of mothers who have seen an obstetrician as their LMC compared to other types of LMC, but there is a need for caution during interpretation of this skewed result as there were few mothers who had seen an obstetrician (only 3%) as their LMC. The proportions of sleep position was similar for all types of LMC.

### Association between determinants of infant care and infant’s sleep position and sleep arrangement

The initial analyses explored the associations between each explanatory variable and an infant’s sleep position and arrangement separately (Table 2). Younger mothers were observed to be more likely to practice sleep arrangements as per guidelines. Other demographic factors such as educational status, and deprivation, were not significantly associated with infant care practices.

**Table 2:** Unadjusted regression evaluating the association between infant care practices and not following guidelines (column %)

*Variables*	*Sleeping position*				*Sleeping arrangements*			
	Yes	No	OR	95% CI	Yes	No	OR	95% CI
Demographic factors								
*Maternal age*								
*Equal or more than 30*	268(40%)	110(38%)	Ref		263(37%)	109(44%)	Ref	
*Less than 30*	400(60%)	183(62%)	1.11	0.84–1.48	442(63%)	136(56%)	**0.74**	0.55–1.00
*Education*								
*No secondary school qualification/Secondary School*	73(11%)	26(9%)	Ref		370(53%)	149(61%)	1.41	0.85–2.35
*Diploma/Trade Cert (NCEA 5–6)*	235(35%)	97(33%)	1.16	0.7–1.92	256(36%)	72(30%)	0.98	0.57–1.69
*Bachelor’s or Higher degree*	358(54%)	168(58%)	1.32	0.81–2.14	77(11%)	22(9%)	Ref	
*Deprivation*								
*Low/Medium (ref)*	136(20%)	55(19%)	Ref		143(20%)	45(6%)	Ref	
*High*	532(80%)	238(81%)	1.11	0.78–1.57	562(80%)	200(28%)	1.13	0.78–1.64
*Maternal mental health and lifestyle*								
*Maternal smoking in pregnancy*								
*Not Smoking*	557(84%)	243(83%)	Ref		576(82%)	215(88%)	Ref	
*Smoking*	110(16%)	49(17%)	1.02	0.71–1.48	129(18%)	28(12%)	0.58	0.38–0.90
*Smoking in same room during pregnancy*								
*Not smoking*	583(87%)	254(87%)	Ref		609(86%)	219(89%)	Ref	
*Smoking*	84(13%)	38(13%)	1.04	0.69–1.57	96(14%)	24(10%)	0.7	0.43–1.12
*Maternal smoking at 9 months*								
*Not smoking*	454(77%)	193(76%)	Ref		472(75%)	168(69%)	Ref	
*Smoking*	139(23%)	61(24%)	1.03	0.73–1.46	155(25%)	44(18%)	0.8	0.55–1.16
*Maternal smoking before pregnancy*								
*Not Smoking*	431(65%)	200(68%)	Ref		453(64%)	174(71%)	Ref	
*Smoking*	236(35%)	92(32%)	0.84	0.63–1.13	252(36%)	69(28%)	**0.71**	0.52–0.98
*Alcohol consumption before pregnancy any* vs *none*								
*No*	281(42%)	137(47%)	Ref		287(41%)	128(52%)	Ref	
*Yes*	387(58%)	156(53%)	0.83	0.63–1.09	418(59%)	117(48%)	**0.63**	0.47–0.84
*Alcohol consumption before pregnancy per week*								
*Did not drink (ref)*	281(42%)	137(47%)	Ref		287(41%)	128(52%)	Ref	
*Less than one drink*	111(17%)	33(11%)	**0.61**	**0.39–0.95**	112(16%)	31(13%)	**0.62**	0.40–0.97
*1–3 drinks*	108(16%)	48(16%)	0.91	0.61–1.36	119(17%)	34(14%)	**0.64**	0.41–0.99
*4+ drinks*	168(25%)	75(26%)	0.92	0.65–1.29	187(27%)	52(21%)	**0.62**	0.43–0.9
*Alcohol consumption 9 m any* vs *none*								
*No*	361(61%)	158(62%)	Ref		363(58%)	151(62%)	Ref	
*Yes*	232(39%)	96(38%)	0.95	0.7–1.28	264(42%)	61(25%)	**0.56**	0.40–0.78
*Alcohol consumption 9 m*								
*Did not drink (ref)*	361(61%)	158(62%)	Ref		363(58%)	151(71%)	Ref	
*Less than one drink*	128(22%)	47(19%)	0.84	0.57–1.23	136(22%)	37(17%)	**0.65**	0.43–0.99
*1–3 drinks*	57(10%)	26(10%)	1.04	0.63–1.72	71(11%)	12(6%)	**0.41**	0.21–0.77
*4+ drinks*	47(8%)	23(9%)	1.12	0.66–1.90	57(9%)	12(6%)	0.51	0.26–0.97
*Maternal Depression*								
*Not depressed*	474(71%)	214(73%)	Ref		498(71%)	185(76%)	Ref	
*Depressed*	194(29%)	79(27%)	0.9	0.66–1.23	207(29%)	60(24%)	0.78	0.56–1.09
Community and cultural context								
*Cultural Connectedness*								
*Very*	456(68%)	217(74%)	Ref		489(69%)	178(73%)	Ref	
*Moderately*	147(22%)	45(15%)	**0.64**	**0.44–0.93**	142(20%)	46(19%)	0.89	0.61–1.29
*Poorly*	65(10%)	30(10%)	0.97	0.61–1.54	74(10%)	20(8%)	0.74	0.44–1.25
*Family support*								
*Very supported*	582(88%)	254(88%)	Ref		520(75%)	165(67%)	Ref	
*Poorly supported*	79(12%)	35(12%)	1.02	0.66–1.55	177(25%)	77(31%)	**1.57**	**1.00–1.89**
*Language*								
*English*	436(65%)	163(56%)	Ref		477(68%)	113(46%)	Ref	
*Not English*	232(35%)	130(44%)	**1.5**	**1.13–1.98**	228(32%)	132(54%)	**2.44**	**1.82–3.29**
Health care access								
*Immunisation intentions*								
*Yes*	625(94%)	275(94%)	Ref		660(94%)	230(94%)	Ref	
*No*	43(6%)	17(6%)	0.9	0.50–1.60	44(6%)	15(6%)	0.98	0.53–1.79
*Family doctor/GP before pregnancy*								
*Yes*	622(93%)	277(95%)	Ref		669(95%)	219(89%)	Ref	
*No*	46(7%)	16(5%)	0.78	0.43–1.4	36(5%)	26(11%)	**2.21**	**1.3–3.74**
*Seen family doctor/GP since pregnant*								
*Yes*	588(88%)	254(87%)	Ref		616(87%)	216(88%)	Ref	
*No*	80(12%)	39(13%)	1.13	0.75–1.7	89(13%)	29(12%)	0.92	0.59–1.45
*Baby’s family doctor/GP*								
*Yes*	555(83%)	236(82%)	Ref		580(83%)	203(84%)	0.96	0.65–1.42
*No*	110(17%)	52(18%)	1.11	0.77–1.6	119(17%)	40(16%)		
*Baby’s family doctor/GP - same as mothers’ pre-pregnancy*								
*Yes*	501(90%)	20(56%)	Ref		532(92%)	181(89%)	Ref	
*No*	54(10%)	16(44%)	0.67	0.38–1.21	48(8%)	22(11%)	1.35	0.79–2.39
*Choice of LMC*								
*Yes*	573(81%)	140(81%)	0.84	0.58–1.21	532(80%)	193(83%)	0.84	0.57–1.24
*No*	138(19%)	33(19%)			131(20%)	40(17%)		
*Length of time to find LMC*								
*Less than 1 week*	321(51%)	130(48%)	Ref		343(54%)	104(50%)	Ref	
*1 to 6 weeks*	225(36%)	109(40%)	1.2	0.88–1.62	244(38%)	85(40%)	1.15	0.83–1.60
*7 to 13 weeks*	46(7%)	17(6%)	0.9	0.50–1.65	37(6%)	11(5%)	1.00	0.48–1.99
*13 weeks or more*	34(5%)	15(6%)	1.09	0.57–2.07	10(2%)	< 10(5%)	1.64	0.55–4.93
*Consulted alternative practitioners stated*								
*Yes*	24(4%)	25(9%)	**2.51**	**1.41–4.48**	40(6%)	< 10(4%)	0.45	0.26–1.22
*No*	644(96%)	267(91%)	Ref		665(94%)	236(96%)	Ref	
*Childbirth preparation*								
*Intended*	103(16%)	39(13%)	1.15	0.63–2.10	103(15%)	35(14%)	1.64	0.84–3.17
*No*	488(74%)	229(79%)	1.43	0.87–2.35	518(74%)	192(79%)	**1.78**	**1.02–3.13**
*Yes*	70(11%)	23(8%)	Ref		77(11%)	16(7%)	Ref	
Others								
*Fully breastfed*								
*Yes*	383(57%)	158(54%)	Ref		397(56%)	140(57%)	Ref	
*No*	285(43%)	135(46%)	1.15	0.87–1.51	308(44%)	105(43%)	0.97	0.72–1.3
*Prematurity*								
*Term*	637(95%)	277(95%)	Ref		674(96%)	231(94%)	Ref	
*Premature*	31(5%)	15(5%)	1.11	0.59–2.09	30(4%)	14(6%)	1.36	0.71–2.61
*Crowding groups*								
*Low*	48(7%)	21(7%)	Ref		48(7%)	21(9%)	Ref	
*Medium*	356(54%)	153(52%)	0.98	0.57–1.7	403(57%)	99(41%)	0.58	0.32–0.98
*High*	260(39%)	119(41%)	1.05	0.6–1.83	252(36%)	123(51%)	1.95	0.64–1.95
*Dwelling*								
*Family ownership*	219(33%)	77(27%)	Ref		233(34%)	59(25%)	Ref	
*Private rental*	258(39%)	117(41%)	1.29	0.92–1.81	284(41%)	89(37%)	1.24	0.85–1.8
*Public rental*	180(27%)	91(32%)	**1.44**	**1.00–2.06**	175(25%)	91(38%)	**2.05**	**1.40–3.01**

*OR = odds ratio, Ref = Reference variable*

In the domain of maternal health-related practices, smoking and alcohol consumption were significantly associated with infants’ sleep arrangement practices. Mothers who reported smoking before pregnancy were more likely to adhere to the specific sleep arrangement guideline. Similar findings were observed for maternal alcohol consumption during pregnancy and when their children were 9-months old.

More than two-thirds of the participants in this study converse in English at home. The mothers who speak English at home were more likely to practice safe infant sleeping positions (65% vs 56%) and safe infant sleeping arrangements (68% vs 46%) compared to mothers who speak other languages. Mothers speaking other languages at home had higher odds of placing an infant in an unsafe sleep position (OR = 1.5, 95% CI:1.13–1.98) or having an unsafe sleeping arrangement (OR = 2.44, 95% CI:1.82–3.29). Another community context, level of family support, was significantly associated with non-adherence to specific sleeping arrangement guidelines as if the mother was poorly supported (OR = 1.57, 95% CI: 1.00–1.89) she was more likely to place the infant in an unrecommended sleep arrangement, whereas the mothers with a moderate level of cultural connectedness were more likely to adhere to sleep position guidelines compared to those with a high level of connectedness (OR = 0.64, 95% CI: 0.44–0.93).

Consulting alternative practitioners rather than a family doctor or GP was associated with being more likely to practice unsafe infant sleeping positions. Other healthcare access factors, such as not having a family doctor or GP before pregnancy (OR = 1.3, 95% CI: 1.30–3.74) and having no intention of attending childbirth preparation (OR = 1.78, 95% CI: 1.02–3.13) were also significantly associated with unsafe infant care practice related to sleeping arrangement.

Other factors, such as crowding and dwelling types, were assessed to determine adherence and non-adherence to specific infant care guidelines. Mothers living in public rentals were more likely to practice unsafe infant sleeping positions (OR = 1.44, 95% CI: 1.00–2.06) and more likely (OR = 2.05, 95% CI: 1.40–3.01) to have unsafe infant sleeping arrangements compared to those living in the house they own.

**Fig. 4:**
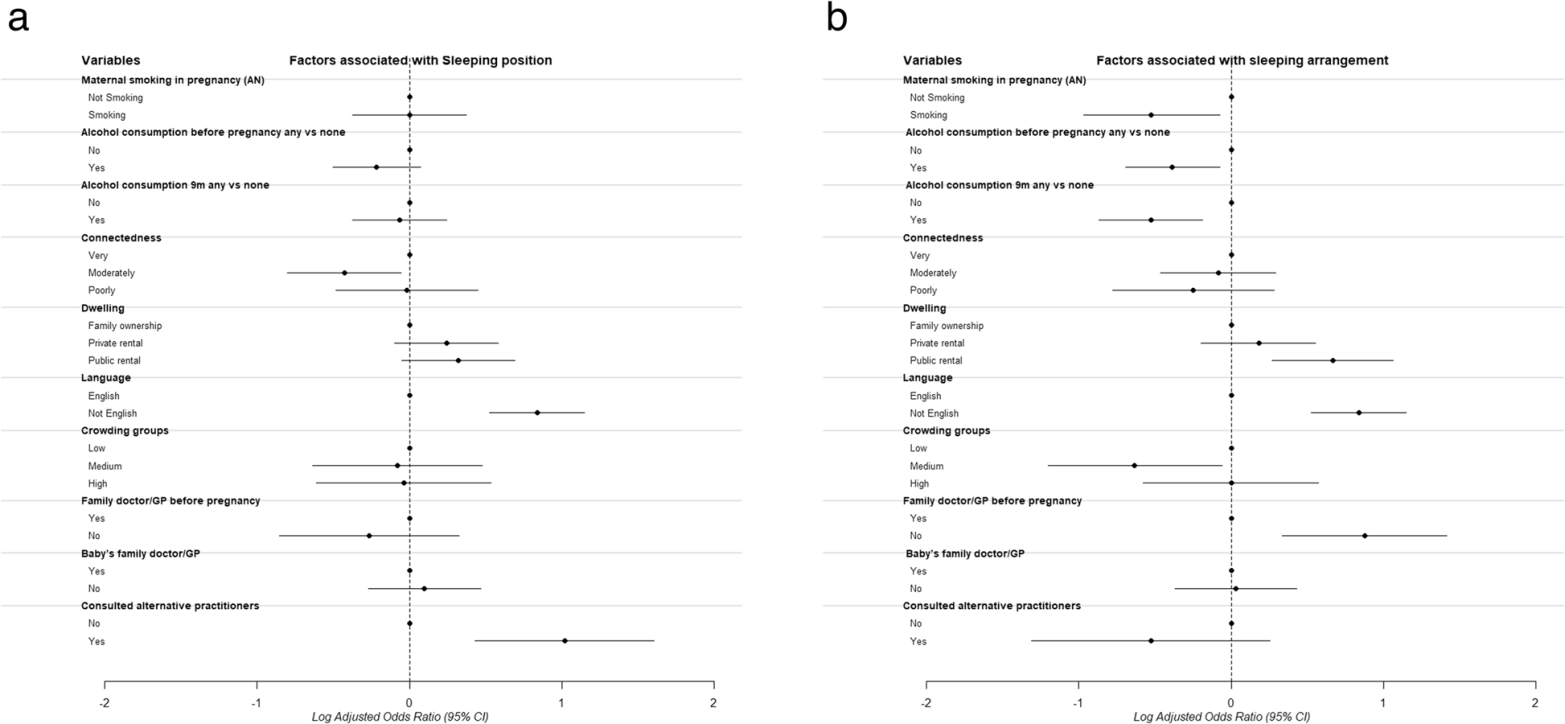
**a** Adjusted analysis evaluating the association between potential determinants and sleeping position. **b**: Adjusted analysis evaluating the association between potential determinants and infant sleeping arrangement

The multivariable analysis adjusted each determinant variable for maternal education, age, and deprivation. In relation to the infants sleeping position (Fig. 4a), only mothers who converse in languages other than English at home and mothers who consulted alternative practitioners had significantly higher odds of not following the guidelines after controlling for basic demographic factors. Being moderately culturally connected versus very culturally connect resulted in lower odds of not meeting sleep guidelines. Smoking and alcohol use were, counterintuitively, associated with lower odds of not following the guidelines for sleeping arrangement. Medium household crowding versus low was associated with lower odds of not following the guidelines. Public rental versus family ownership, non-english speaking and not having a family doctor prior to pregnancy had higher odds of not following the guidelines for sleeping arrangement, (Fig. 4b). Expected family support and intention to attend preparation of childbirth education were not significant in the final adjusted model for sleep arrangements.

## Discussion

In this study exploring infant care practices for Pacific mothers in the Growing Up in New Zealand study just over two thirds of mothers reported their babies slept in a separate cot/bassinet in their parents’ room and just under two thirds reported sleeping their babies on their backs as per infant safe sleep guidelines. Most mothers had a midwife as their LMC. Unadjusted associations were observed between maternal age, smoking and alcohol consumption, language spoken at home, family support, access to a GP before pregnancy, childbirth preparation classes and housing tenure with adherence to sleep arrangement guidelines. Similarly, unadjusted associations were observed between alcohol consumption, level of cultural connectedness, language spoken at home, use of alternative practitioners, and housing tenure with adherence to sleep position guidelines. After adjusting for demographic factors, associations remained for language, connection to culture, consultations with an alternative practitioner and sleep position, and language, smoking, alcohol, dwelling, crowding and access to the family doctor or GP were associated with infant sleeping arrangement practices. Our findings indicate that safe sleep messaging is not reaching or connecting with Pacific mothers who: do not speak English at home, use alternative health practitioners and are more socioeconomically disadvantaged, but it is reaching those who are moderately culturally connected and who live in a larger household.

This is the first time the association between a wide range of social, demographic and environmental factors and Pacific infant care practises have been investigated. A Pacific focus was important because of the disproportionate representation of Pacific infants and families impacted by SUDI and GUiNZ is a contemporary longitudinal dataset, which provides some generalisability to the Aotearoa New Zealand population [26]. However, the infant care practice responses probably represent what parents aspire to do most of the time. The survey won’t capture variation in infant care practices which might occur when the routine is disrupted such as holidays, social, wider family circumstances and how often these disruptions occur. Logistic regression modelling was used because it is a robust method to examine associations and confounding with a dichotomous outcome.

Pacific mothers who do not speak English at home, or use alternative health practitioners were less likely to follow guidelines, highly likely due to the guidelines either 1. Not reaching them, or 2. They might be just translations of the English and lack a cultural world view which may be important for justifying or comprehending why, or 3 historical poor experiences in the health system diminishing trust in the messaging, or 4. All of the above. Racism at individual, systemic and societal levels, is well recognised to negatively impact on health and wellbeing and contribute to inequities [37] and experiences of racism by a health professional have been found to be substantially higher for Pacific when compared to European [38]. Conversely, mothers who were moderately culturally connected or lived in homes with moderate crowding (larger households) were more likely to follow guidelines. This confirms the protective nature of being connected to culture, alongside confirmation of previous findings showing larger households during COVID-19 correlated with a reduction in depression in this same cohort [39].

The counter-intuitive finding related to a reversed association of alcohol and smoking with infantcare practices was not unexpected, as this has been found previously in other health research. A similar pattern was found when looking at the SDQ data related to lower drinking levels in *GUiNZ* at 8 years [40, 41]. Mild exposure to alcohol and smoking may in fact be acting as a proxy for socioeconomic status and a marker for social connectedness. It may also be that those who acknowledge exposure to alcohol and smoking prenatally are more likely to be given information on the infant guidelines and the risks related to alcohol and smoking. It is important to specify that this study investigates the relationship between risk factors related to SUDI, and infant sleeping arrangements and position, and therefore the likelihood of co-occurrence, not SUDI as an outcome. When other studies have investigated actual SUDI deaths there has been a definite link between SUDI and alcohol and/or smoking [4, 42, 43].

Lower socioeconomic status and poverty are underlying factors in many aspects of health, and more stark for Pacific due to the greater inequities in social determinants. A 2020 Ministry of Health report found less than 20% of SUDI whānau were living without considerable financial insecurity, and in most cases, families were living in shared accommodation, boarding, renting and living in one room. Poverty and lack of adequate, affordable housing are likely barriers for ensuring there are safe sleeping arrangements, and also have implications for the wellbeing of carers to provide optimal care [17].

In terms of healthcare providers, longstanding relationships and access to primary care have in other studies, also been found important for improving engagement with services and decision making around infant sleep environments [44].

Understanding Pacific views of sleep is important, and a qualitative study which did this found the dominant discourse around sleep interventions ‘*rarely accounts for cultural variations and contexts which fall outside these approaches* [45]. Their themes related to family in motion, physical closeness, economic pressures, family, community, culture and faith. The concept of solitary sleeping is the exception not the rule due to strong values of interconnectedness. Oversimplifying or overlooking these understandings of healthy sleep risk a disconnect with safe sleep messaging.

Clinicians and practitioners must continue to emphasise the importance of safe infant sleep environments, however in a way that takes time to understand reasons for non-adherence and is able to interweave cultural frameworks and understandings [8, 15]. Most importantly a paradigm shift must occur, moving from placing responsibility on families, to recognising the responsibility of health professionals, providers and the system to provide fit for purpose care to those who need it most [17]. This includes a deeper exploration of the stigmas associated with SUDI, and how to dispel myths including the innuendo of neglect [8].

Affordable and appropriate safer sleep spaces for infants are important in preventing SUDI. They also reinforce the safe sleep messaging [17]. Wahakura (bassinet shaped flax basket) and Pepi-pods (shallow plastic box) have been developed and provided in Aotearoa New Zealand and offered to mothers at higher risk of SUDI [4, 46, 47].

A review of New Zealand’s National SUDI Prevention Programme (NSPP) found that it is incohesive, and lacks coordination, systems leadership and strategic direction across the key partners [48]. Health promotion and preventative campaigns continue to be important, however not at the expense of addressing equitable access to care, social determinants of health and racism [7].

A comprehensive set of recommendations for infants, parents, health care providers, the health system, and researchers, place a high burden of responsibility on decision makers to provide more resources for improving inequities, and shift power to Pacific communities, experts and leaders to improve SUDI outcomes [5]. This call has been reinforced in the recommendations by Tipene-Leach and Fidow [17] to align with Te Aka Whai Ora (Māori Health Authority), prioritise a Māori and Pacific framework and leadership, follow a Hauora wellbeing approach and ensuring solutions are culturally anchored and whānau/aiga led and delivered in partnership with community providers.

In 2002, 62% of Pacific mothers were able to identify at least one risk factor for SUDI [49]. A 2020 New Zealand Ministry of Health report on SUDI knowledge related to 64 infant deaths between 2019 and 2020 [17] found that while safe sleep messages are heard, acting on the knowledge is limited. SUDI interventions have shown improvement in knowledge [50], however understanding what is required to shift knowledge to practise, particularly for Pacific families, requires further investigation [9, 14]. A second stage of this study is a qualitative exploration of research needed for the development of a culturally appropriate intervention and communication strategy for reducing the risk of SUDI amongst Samoan, Tongan and Cook Islands Māori and Niuean mothers in New Zealand and a case study of Cook Islands and Niue mothers in their home Islands.

The link between inequities for Pacific infants and SUDI, and experiences of racism is an important aspect that wasn’t explored in this study and is needed. Already it is well known that SUDI is one of the most underfunded areas of paediatric research [51].

## Conclusion

Aotearoa New Zealand has a long history when it comes to equity of experience in healthcare, no less so when it comes to infants and families who experience SUDI. Because of this, Pacific families must be prioritised when it comes to the distribution of resources, and development of interventions related to SUDI in Aotearoa New Zealand. This study confirms the need for diverse perspectives on infant care and Pacific paradigms and frameworks to be incorporated to combat historical, systemic and individual injustices.

## Data Availability

The data that support the findings of this study are available from the Growing Up in New Zealand study [please see https://www.growingup.co.nz/access-growing-data, or contact data access co-ordinator at “dataaccess@growingup.co.nz“], but restrictions apply to the availability of these data, which were used under license for the current study, and so are not publicly available. The data are not publicly available due to containing information that could compromise research participant privacy and consent.
